# Clinical efficacy of stereotactic ablative radiotherapy for lung metastases arising from colorectal cancer

**DOI:** 10.1186/s13014-015-0546-x

**Published:** 2015-11-21

**Authors:** Jinhong Jung, Si Yeol Song, Jong Hoon Kim, Chang Sik Yu, Jin Cheon Kim, Tae Won Kim, Seong-Yun Jeong, Su Ssan Kim, Eun Kyung Choi

**Affiliations:** Department of Radiation Oncology, Asan Medical Center, University of Ulsan College of Medicine, 88, Olympic-ro 43-gil, Songpa-gu, Seoul, 05505 Republic of Korea; General Surgery, Asan Medical Center, University of Ulsan College of Medicine, Seoul, Republic of Korea; Internal Medicine Asan Medical Center, University of Ulsan College of Medicine, Seoul, Republic of Korea; Asan Institute for Life Science, Asan Medical Center, University of Ulsan College of Medicine, Seoul, Republic of Korea; Present address: Department of Radiation Oncology, Kyung Hee University Medical Center, Kyung Hee University School of Medicine, Seoul, Republic of Korea

**Keywords:** Stereotactic ablative radiotherapy, Lung metastases, Colorectal cancer

## Abstract

**Background:**

Limited data describe the prognosis after stereotactic ablative radiotherapy for lung metastases arising from colorectal cancer. Thus, we evaluated treatment outcomes of stereotactic ablative radiotherapy for those patients.

**Methods:**

The study involved patients received stereotactic ablative radiotherapy for one to three lung metastases arising from colorectal cancer at a single institution. A total dose of 40–60 Gy (median, 48 Gy) in three or four fractions was prescribed.

**Results:**

A total of 79 metastatic lung lesions from 50 patients who underwent curative resection for their primary colorectal cancer or salvage treatment at a recurrent site were included. The one- and three-year local control rates were 88.7 % and 70.6 %, respectively. The three-year overall survival and progression-free survival rates were 64.0 % and 24.0 %, respectively. Patients with tumor volume ≤1.5 mL had a significantly better overall survival rate than those with tumor volume >1.5 mL (68.0 % vs. 60.0 % at three-year, *p* = 0.02). Local control was associated with a trend towards better survival (*p* = 0.06). No pulmonary complications greater than grade 2 were observed.

**Conclusion:**

Stereotactic ablative radiotherapy is a competitive treatment modality for the management of lung metastases arising from colorectal cancer.

## Background

The lung is one of the most likely sites of metastases in the majority of solid-organ cancers [1]. In colorectal cancer (CRC), the frequency of lung metastases is second only to that in the liver [2, 3]. The incidence of lung metastases associated with CRC is typically reported to be 8–10 % over the life of the affected patient [2–4]. As for oligometastases to the liver, lung metastases in CRC are considered to have been eradicated if the metastatic tumor burden is relatively low (usually 1 to 3). This consensus was derived from encouraging treatment results following salvage resection of lung metastases over the past several decades. Pulmonary metastasectomy, a procedure introduced by Weinlechnerin in 1882, has been shown to improve survival in these patients [5]. The 5-year overall survival (OS) rates of patients undergoing resection of pulmonary metastases arising from CRC are in the range of 24–62.7 % for R0 resections and 0–21 % for R1 resections [6–8]. However, only about 10 % of all patients with clinically isolated metastases to the lung from a primary CRC are candidates for pulmonary metastasectomy because of their medical condition or refusal to undergo surgery [9]. Patients with untreated metastatic disease have a 5-year survival rate of less than 5 %, with a median survival time of 10 months [10, 11].

Stereotactic ablative radiotherapy (SABR) is a good option for patients with pulmonary oligometastases who are medically inoperable or refuse to undergo surgery. The use of SABR has increased recently due to the recognition that it is a safe and effective treatment for patients with fewer than four lung metastases [1, 12–14]. Rusthoven et al. reported that actuarial local control (LC) rates one and two years after SABR of 48–60 Gy for pulmonary oligometastases were 100 % and 96 %, respectively, with local progression in only one patient [14].

To date, only one study has focused on the feasibility of SABR for isolated colorectal lung metastases. Kim et al. investigated the effectiveness of SABR for the treatment of 18 lesions in 13 patients, and reported three-year OS, LC, and progression-free survival (PFS) rates of 64.7 %, 52.7 %, and 11.5 %, respectively [15]. Unfortunately however, this study included too few patients to permit effective comparisons of the reported survival outcomes with a surgical series. In our current study, we report the efficacy of SABR for lung metastases arising in patients with controlled primary CRC, with homogeneous data generated from a single radiation oncology unit. Our evaluation of treatment outcomes included not only LC, but also the OS and PFS rates.

## Materials and methods

### SABR for lung metastases

Our institution has developed guidelines for the indications of SABR for lung metastases arising from lung or other primary-organ cancers. All patients who were included in our current retrospective study were treated in accordance with these strict guidelines at a single institution between January 2003 and January 2011. We performed SABR for the treatment of lung metastases from CRC only if a patient satisfied all of the following criteria: (1) no evidence of recurrence at the primary site; (2) no extrapulmonary metastasis or other primary cancer; (3) no mediastinal node metastasis judged by computed tomography (CT) and positron emission tomography/computed tomography (PET/CT); (4) a tumor size smaller than 5 cm at its longest diameter, with fewer than four metastatic nodules; (5) no prior thoracic irradiation; (6) an Eastern Cooperative Oncology Group (ECOG) performance status of 0–2; (7) no active systemic, pulmonary, or pericardial infection; (8) adequate hematologic function (an absolute neutrophil count of >1,500 cells/mm^3^, and a platelet count of >100,000 cells/mm^3^); and (9) refusal of invasive surgery, unfit for limited resection, or medical problem(s) including old age or underlying chronic disease. This study was approved by the Institutional Review Board of Asan Medical Center, and informed consent was waived due to the retrospective nature of this study.

### SABR technique

Each patient was immobilized in the supine position, with arms above the head with a vacuum pillow using a stereotactic body frame (Elekta, Stockholm, Sweden). Simulation was done using radiation oncology–dedicated CT (GE LightSpeed RT 16; Healthcare, Waukesha, WI) with shallow free breathing using a 4D-respiratory gating system (RPM™, Varian Medical Systems, Palo Alto, CA). When the treatment site was in the posterior region, the prone position was selected. All CT images were obtained using a 2.5-mm slice thickness. The gross tumor volume (GTV) delineated the visible gross tumor at lung window setting on the end expiratory phase CT image and the internal tumor volume (ITV) was expanded by respiratory movement. The planning target volume (PTV) margin was 5 mm from the ITV. All treatments were planned and administered using four to eight coplanar and/or non-coplanar beams generated by a linear accelerator with energies of 6 to 15 MV. A total dose of 40–60 Gy in three or four fractions to the isodose line covering the PTV (generally the 85–90 % of the isodose line) was prescribed according to the physician’s judgment with consideration of the patient’s general condition, PTV, and radiation dose delivered to normal organs. Contouring and treatment planning were developed using a 3D radiotherapy planning system (Eclipse V8.0; Varian Medical Systems, Palo Alto, CA).

Prior to July 2006, all patients were reviewed offline with CT simulation every other day. Thereafter, cone-beam CT was performed for image guidance for every treatment and radiation was administered using LINAC (CL21iX; Varian Medical Systems, Palo Alto, CA) equipped with an On-Board Imager (Varian Medical Systems).

### Follow-up evaluation after SABR

During SABR, a physician evaluated each patient every day. After SABR, patients had follow-up examinations every 1–3 months consisting of physical examination, blood tests (complete blood count, chemistry, and carcinoembryonic antigen (CEA) level), chest CT, abdomen CT, and/or PET/CT. The initial tumor response was assessed using Response Evaluation and Criteria for Solid Tumors (RECIST version 1.1) four weeks after SABR. Local progression was defined as new or progressive lesions arising in the radiation field. Lesions within radiation pneumonitis or fibrosis that did not change in size were regarded as an indication of stable disease. Lesions arising outside the radiation field within the lung were defined as out-of-field lung progression. Distant progression was defined as recurrent disease at any site outside the lung. Intrathoracic lymph node metastasis was also regarded as a distant progression. Acute toxicities (radiation pneumonitis, esophagitis, skin reaction, rib fracture, and hematologic toxicity) were defined as toxicities that occurred within six months of the completion of SABR and were graded according to the Common Terminology Criteria for Adverse Events (CTCAE; version 4.0).

### Statistical analysis

LC and survival rates were calculated from the last day of SABR. The probability of cumulative LC and survival was determined using the Kaplan-Meier method. Group comparisons were analyzed by the log-rank test. These analyses were performed using SPSS (version 12.0; SPSS Inc., Chicago, IL, USA).

## Results

A total of 79 metastatic lung lesions from 50 patients who had undergone curative resection for their primary CRC or salvage treatment at a recurrent site were treated with SABR. Twenty-seven of these patients had a solitary lesion, 17 had two lesions, and six had three lesions. The median age was 65 years (range, 30–82 years), and 40 (80.0 %) patients had an ECOG PS score of 0–1. Of the 50 patients in the study cohort, 39 (78.0 %) were treated using SABR for metachronous lung metastases and 11 (22.0 %) for synchronous lung metastases. Thirty two (64.0 %) cases were inoperable because of old age or medical problems, and 18 (36.0 %) refused surgical treatment. Eighteen (36.0 %) patients, including 11 with synchronous lung metastases, did not receive prior chemotherapy before SABR. Patient and tumor characteristics are summarized in Table 1.

**Table 1:** Patient and tumor characteristics

Variables	No. of patients (%)
Age (years)	
Median	65
Range	30–82
ECOG performance score	
0	6 (12.0)
1	34 (68.0)
2	10 (20.0)
Timing of SABR	
Synchronous	11 (22.0)
1^st^ recurrence	22 (44.0)
2^nd^ recurrence	14 (28.0)
3^rd^ recurrence	3 (6.0)
Previous Chemotherapy before SABR	
Yes	32 (64.0)
No	18 (36.0)
Reason for SABR	
Inoperable	32 (64.0)
Other	18 (36.0)
GTV (mL)	
Median	1.5
Range	0.2–34.8
Prescription dose	
40 Gy / 4 fx	1 (1.3)
48 Gy / 4 fx	55 (69.6)
60 Gy / 4 fx	20 (25.3)
60 Gy / 3 fx	3 (3.8)

*ECOG* Eastern Cooperative Oncology Group, *SABR* stereotactic ablative body radiotherapy, *GTV* gross tumor volume, *fx* fraction

### Tumor & SABR characteristics

The median GTV of the 79 lesions was 1.5 mL (range, 0.2–34.8 mL). We prescribed 48 Gy in 4 fractions for 55 lesions, 60 Gy in 4 fractions for 20 lesions, 60 Gy in 3 fractions for 3 lesions, and 40 Gy in 4 fractions for 1 lesion (Table 1).

### Tumor response and local control

**Fig. 1:**
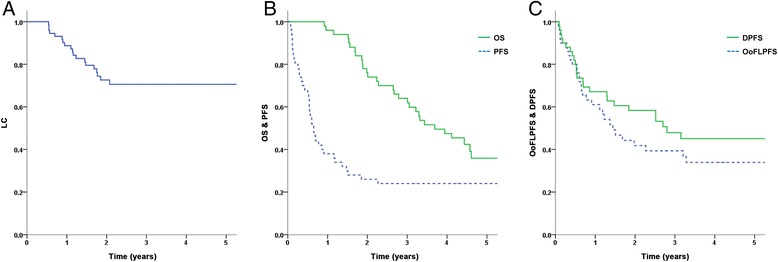
**a** Local control. **b** Overall survival and progression-free survival. **c** Out-of-field lung progression-free survival and distant progression-free survival

The median follow-up period was 42.8 months (range, 11.0–104.1 months). The complete response, partial response, and stable disease rates were 3.8 %, 26.6 %, and 69.6 %, respectively. The one- and three-year LC rates were 88.7 % and 70.6 %, respectively (Fig. 1a).

### Survival outcomes

The three-year actuarial OS and PFS rates were 64.0 % and 24.0 %, respectively (Fig. 1b). The three-year out-of-field lung progression-free survival and distant progression-free survival rates were 39.4 % and 47.9 %, respectively (Fig. 1c).

### Patterns of failure

Local progression as the first site of progression occurred in five patients, out-of-field lung progression in 20 patients, and distant progression in 12 patients. Local, out-of-field lung, and distant progression occurred in one patient. Among these, 22 patients received further chemotherapy.

### Toxicities

Only two (4 %) patients experienced grade 2 pulmonary toxicity (pneumonitis), and there were no grade 3 or greater complications in our patient cohort.

### Factors affecting the treatment outcomes

**Fig. 2:**
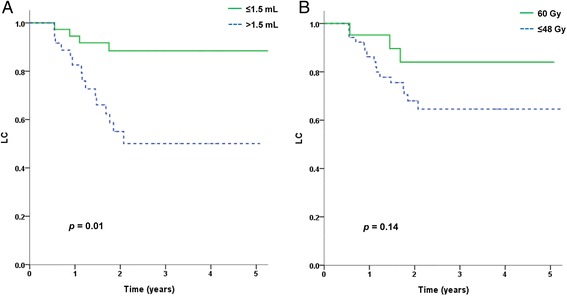
Local control according to **a** GTV and **b** prescribed dose

The results of univariate analyses indicated that the pre-SABR level of CEA (within normal range; *p* < 0.01), GTV (less than 1.5 mL; *p* = 0.01), and timing of SABR (for a second or third pulmonary metastasis; *p* = 0.04) were statistically significant prognostic factors for LC (Table 2, Fig. 2a). A SABR dose of 60 Gy showed a trend towards better LC (*p* = 0.14) (Table 2, Fig. 2b). Local progression was observed in only three of 23 cases who received a dose of 60 Gy.

**Table 2:** Univariate analysis of prognostic factors associated with the local control and overall survival

Factors	n	3-year LC (%)	*p* value	n	3-year OS (%)	*p* value
Age (years)			0.44			0.80
<65	35	73.3		24	58.3	
≥65	44	67.5		26	69.2	
ECOG performance score			0.43			0.47
0–1	66	71.6		40	70.0	
2	12	62.3		9	33.3	
Stage of the primary tumor			0.98			0.43
Stage 1–2	20	69.4		13	61.5	
Stage 3–4	57	70.9		35	65.7	
Timing of SABR			0.04			0.42
First pulmonary metastasis	51	62.6		33	72.7	
Second or third	28	90.9		17	47.1	
Pre-SABR CEA level			<0.01			0.40
≤6	62	78.1		39	64.1	
>6	10	0		6	66.7	
GTV			0.01			0.02
≤1.5 mL	41	88.5		25	68.0	
>1.5 mL	38	50.1		25	60.0	
Radiation dose			0.14			0.12
≤48 Gy	56	64.6		33	54.5	
60 Gy	23	84.0		17	82.4	
LC			-			0.06
Yes	-	-		39	66.7	
No	-	-		11	54.5	

*LC* local control, *OS* overall survival, *ECOG* Eastern Cooperative Oncology Group, *SABR* stereotactic ablative body radiotherapy, *CEA* carcinoembryonic antigen, *GTV* gross tumor volume

**Fig. 3:**
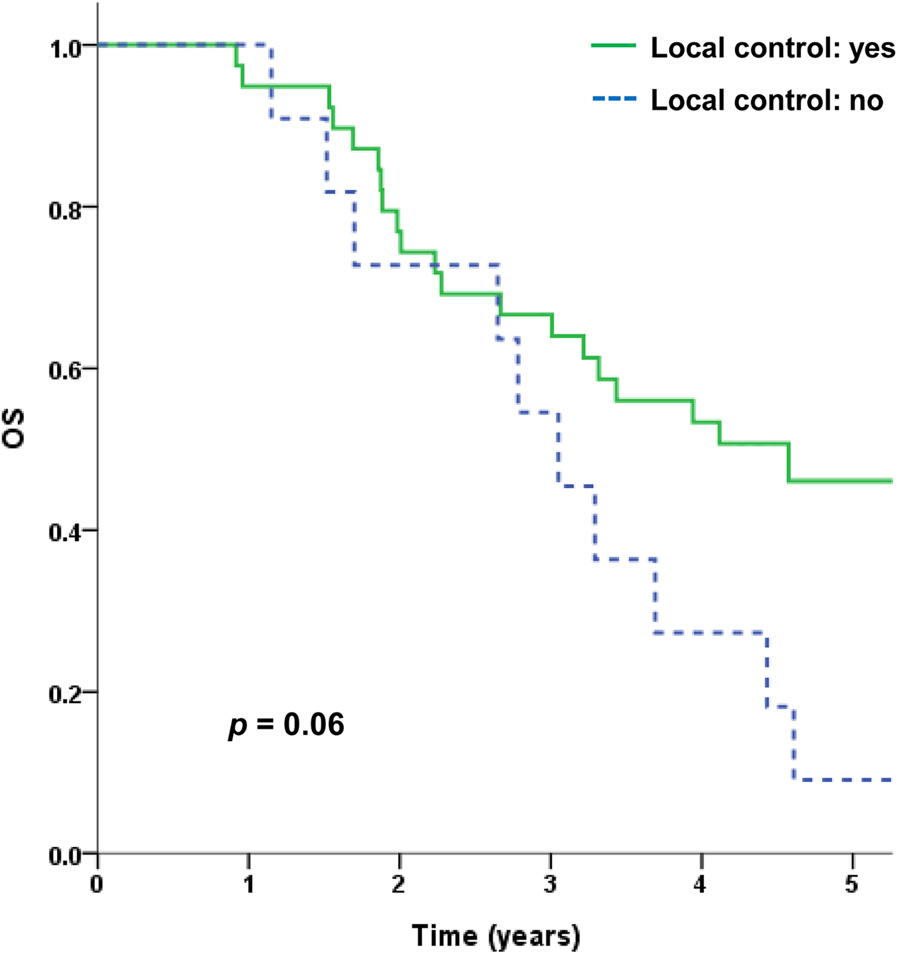
Overall survival according to local control

OS was significantly better according to GTV. Patients with tumor volume ≤1.5 mL had a better OS rate than those with tumor volume >1.5 mL (Table 2). Local control was associated with a trend towards better survival (Table 2, Fig. 3).

## Discussion

SABR is one of several effective treatment options for patients with pulmonary oligometastases that are medically inoperable or who refuse to undergo surgery. Several studies have concluded that SABR is a safe and effective treatment for patients with oligo-lung metastases [1, 12–14]. Rusthoven et al. performed a multi-institutional phase I, II trial of SABR for pulmonary oligometastases from a variety of primary sites [14]. The study cohort included nine patients with CRC, and the authors reported actuarial LC rates of 100 % and 96 % at one- and two years after SABR with 48–60 Gy, respectively, and a two-year OS rate of 39 %. Local progression occurred in only one patient with a high-grade extremity sarcoma and no cases of grade 4 or 5 toxicity were observed. Norihisa et al. evaluated their experience with SABR of 48 Gy or 60 Gy (12 Gy per fraction) in the treatment of oligometastatic lung tumors from several origins in a total of 34 patients, including nine cases of CRC. This study reported OS, local relapse-free, and progression-free rates at two years of 84.3 %, 90.0 %, and 34.8 %, respectively [13]. Recently, Filippi et al. studied to estimate SABR efficacy and its potential role as an alternative to surgery for the treatment of lung metastases from CRC [16]. Forty consecutive patients who received SABR as first local therapy were analyzed, two-year OS rate was 73 % and failure at SABR site was observed in 3 patients (7.5 %).

In our present study, the three-year actuarial LC rate was 70.6 %. In addition, the three-year LC rate of 82.4 % for patients receiving 60 Gy was comparable with that reported in previous studies [1, 12–17]. Subgroup analysis revealed that a GTV smaller than 1.5 mL indicated better local control than a GTV larger than 1.5 mL. The CEA level prior to SABR was another prognostic factor for LC. We assume that the tumors with an elevated CEA level are more aggressive in nature and that tumor control is more difficult than for tumors with a CEA level in the normal range when using the same prescription dose. Furthermore, although LC between 60 Gy and below 48 Gy was not statistically significant (*p* = 0.14), the higher dose may be appropriate to control CRC metastasis to the lungs (Table 2, Fig. 2b). However, because the present study is limited in terms of events of local progression, further study is required to define a higher dose effect for local control.

A major pattern of failure was found to result from out-of-field lung progression in our current patient cohort. Out-of-field lung progression arose at a rate of 60.6 %, and distant progressions at a frequency of 52.1 % at three years after SABR (Fig. 1c). Among 38 patients who experienced recurrence, 20 had out-of-field lung progressions as the first site of progression. Oligometastases were reported for 11 of these 20 patients, and multiple metastases for another nine patients. Among the 11 patients with oligometastases in our study cohort, ten (50 % of the total patients with out-of-field lung progression as the first site of progression) received salvage local treatment, which involved wedge resection and/or SABR. Half of these patients are currently alive with stable disease (Table 3).

**Table 3:** Salvage treatment for patients with an out-of-field lung progression as the first site of progression

Treatment modality (total 20 patients)	n (%)	Final disease status	
		Stable disease	Progressive disease
SABR	8 (40)	3	5
Wedge Resection	2 (10)	2	0
Chemotherapy only	8 (40)	3	5
No further treatment	2 (10)	0	2

*SABR* stereotactic ablative body radiotherapy

The three- and five-year actuarial OS rates were 64.0 % and 35.9 %, respectively. Although the follow-up period was not long enough and the patient and tumor characteristics were quite different between surgical series and this study, making direct comparison challenging, the OS in this study could be competitive with previous surgical series that showed a 5-year OS for patients undergoing resection of pulmonary metastases in the range of 24–62.7 % for R0 resections and 0–21 % for R1 resections. Pfannschmidt et al., in their systematic review of surgical resection of pulmonary metastases from CRC, demonstrated that CEA level before treatment was the most consistently reported potential prognostic indicator for OS [6]. In studies of surgery, approximately 40–47 % of the study cohorts had higher CEA levels than normal [7, 8, 18–20]. However, the results of our present study included too few patients with high CEA levels (6 patients, 13.9 %) to demonstrate a statistically significant difference in OS rates. Kim et al. have demonstrated LC and OS rates of patients with inclusion criteria for SABR that were similar to the patients in our present study [15]. In their study, 13 patients (18 lesions) received SABR with doses in the range of 39–51 Gy in three fractions and showed a 3-year LC rate of 52.7 % and an OS rate of 64.7 %. The total ITV tended to be associated with OS. In our present study, GTV was found to be a statistically significant prognostic factor for LC and OS. In addition, the effect of LC on OS was found to be marginally significant (*p* = 0.06).

This study has some inherent limitations due to its retrospective design, including selection bias, numerous treatment modalities and a short follow-up period. Nevertheless, we utilized a relatively large and homogeneous study cohort of patients with controlled primary CRC who received SABR for lung metastases. We observed promising local control and comparable survival to that of other local treatment modalities. Further large-scale studies are needed to compare treatment modalities and define indications for nonsurgical local treatment modalities for lung metastasis from CRC.

In summary, we investigated treatment outcomes of SABR for 79 metastatic lung lesions from 50 patients who underwent curative resection for their primary CRC or salvage treatment at a recurrent site. The one- and three-year LC rates were 88.7 % and 70.6 %, respectively. The three-year OS and PFS rates were 64.0 % and 24.0 %, respectively. SABR is a feasible, safe, and effective treatment modality and it should be considered a competitive treatment modality for the management of lung metastasis arising from CRC.

## Abbreviations

CEA: carcinoembryonic antigen
CRC: colorectal cancer
CT: computed tomography
ECOG: Eastern Cooperative Oncology Group
GTV: gross tumor volume
ITV: internal tumor volume
LC: local control
OS: overall survival
PET/CT: positron emission tomography/computed tomography
PFS: progression-free survival
PTV: planning target volume
SABR: stereotactic ablative radiotherapy

## References

[1] Okunieff P, Petersen AL, Philip A, Milano MT, Katz AW, Boros L (2006). Stereotactic Body Radiation Therapy (SBRT) for lung metastases. Acta Oncol.

[2] August DA, Ottow RT, Sugarbaker PH (1984). Clinical perspective of human colorectal cancer metastasis. Cancer Metastasis Rev.

[3] McCormack PM, Burt ME, Bains MS, Martini N, Rusch VW, Ginsberg RJ (1992). Lung resection for colorectal metastases. 10-year results. Arch Surg.

[4] Goya T, Miyazawa N, Kondo H, Tsuchiya R, Naruke T, Suemasu K (1989). Surgical resection of pulmonary metastases from colorectal cancer. 10-year follow-up. Cancer.

[5] Weinlechner J (1882). Zur Kasuistik der Tumoren der Brustwand und deren Behandlung (Resektion der Rippen, Eroffnung der Brusthohle, partielle Entfernung der Lunge). Wien Med Wochenschr.

[6] Pfannschmidt J, Dienemann H, Hoffmann H (2007). Surgical resection of pulmonary metastases from colorectal cancer: a systematic review of published series. Ann Thorac Surg.

[7] Melloni G, Doglioni C, Bandiera A, Carretta A, Ciriaco P, Arrigoni G (2006). Prognostic factors and analysis of microsatellite instability in resected pulmonary metastases from colorectal carcinoma. Ann Thorac Surg.

[8] Rena O, Casadio C, Viano F, Cristofori R, Ruffini E, Filosso PL (2002). Pulmonary resection for metastases from colorectal cancer: factors influencing prognosis. Twenty-year experience. Eur J Cardiothorac Surg.

[9] Pihl E, Hughes ES, McDermott FT, Johnson WR, Katrivessis H (1987). Lung recurrence after curative surgery for colorectal cancer. Dis Colon Rectum.

[10] Seymour MT, Stenning SP, Cassidy J (1997). Attitudes and practice in the management of metastatic colorectal cancer in Britain. Colorectal Cancer Working Party of the UK Medical Research Council. Clin Oncol (R Coll Radiol).

[11] Simmonds PC (2000). Palliative chemotherapy for advanced colorectal cancer: systematic review and meta-analysis. Colorectal Cancer Collaborative Group. BMJ.

[12] Lee SW, Choi EK, Park HJ, Ahn SD, Kim JH, Kim KJ (2003). Stereotactic body frame based fractionated radiosurgery on consecutive days for primary or metastatic tumors in the lung. Lung Cancer.

[13] Norihisa Y, Nagata Y, Takayama K, Matsuo Y, Sakamoto T, Sakamoto M (2008). Stereotactic body radiotherapy for oligometastatic lung tumors. Int J Radiat Oncol, Biol, Phys.

[14] Rusthoven KE, Kavanagh BD, Burri SH, Chen C, Cardenes H, Chidel MA (2009). Multi-institutional phase I/II trial of stereotactic body radiation therapy for lung metastases. J Clin Oncol.

[15] Kim MS, Yoo SY, Cho CK, Yoo HJ, Choi CW, Seo YS (2009). Stereotactic body radiation therapy using three fractions for isolated lung recurrence from colorectal cancer. Oncology.

[16] Filippi AR, Badellino S, Ceccarelli M, Guarneri A, Franco P, Monagheddu C (2015). Stereotactic ablative radiation therapy as first local therapy for lung oligometastases from colorectal cancer: a single-institution cohort study. Int J Radiat Oncol, Biol, Phys.

[17] Jung IH, Song SY, Jung J, Cho B, Kwak J, Je HU (2015). Clinical outcome of fiducial-less CyberKnife radiosurgery for stage I non-small cell lung cancer. Radiation oncology journal.

[18] Iizasa T, Suzuki M, Yoshida S, Motohashi S, Yasufuku K, Iyoda A (2006). Prediction of prognosis and surgical indications for pulmonary metastasectomy from colorectal cancer. Ann Thorac Surg.

[19] Pfannschmidt J, Muley T, Hoffmann H, Dienemann H (2003). Prognostic factors and survival after complete resection of pulmonary metastases from colorectal carcinoma: experiences in 167 patients. J Thorac Cardiovasc Surg.

[20] Saito Y, Omiya H, Kohno K, Kobayashi T, Itoi K, Teramachi M (2002). Pulmonary metastasectomy for 165 patients with colorectal carcinoma: A prognostic assessment. J Thorac Cardiovasc Surg.

